# Screw Osteointegration—Increasing Biomechanical Resistance to Pull-Out Effect

**DOI:** 10.3390/ma16165582

**Published:** 2023-08-11

**Authors:** Bogdan Costăchescu, Adelina-Gabriela Niculescu, Alexandru Mihai Grumezescu, Daniel Mihai Teleanu

**Affiliations:** 1 “Gr. T. Popa” University of Medicine and Pharmacy, 700115 Iasi, Romania; costachescus@gmail.com; 2 “Prof. Dr. N. Oblu” Emergency Clinical Hospital, 700309 Iasi, Romania; 3 Research Institute of the University of Bucharest—ICUB, University of Bucharest, 050657 Bucharest, Romania; adelina.niculescu@upb.ro; 4 Department of Science and Engineering of Oxide Materials and Nanomaterials, Politehnica University of Bucharest, 011061 Bucharest, Romania; 5 Academy of Romanian Scientists, Ilfov No. 3, 050044 Bucharest, Romania; 6 “Carol Davila” University of Medicine and Pharmacy, 050474 Bucharest, Romania; daniel.teleanu@umfcd.ro

**Keywords:** bone fixation, spinal fixation devices, spinal screws, pull-out effect, reinforcement strategies, osteointegration, material optimization

## Abstract

Spinal disorders cover a broad spectrum of pathologies and are among the most prevalent medical conditions. The management of these health issues was noted to be increasingly based on surgical interventions. Spinal fixation devices are often employed to improve surgery outcomes, increasing spinal stability, restoring structural integrity, and ensuring functionality. However, most of the currently used fixation tools are fabricated from materials with very different mechanical properties to native bone that are prone to pull-out effects or fail over time, requiring revision procedures. Solutions to these problems presently exploited in practice include the optimal selection of screw shape and size, modification of insertion trajectory, and utilization of bone cement to reinforce fixation constructs. Nevertheless, none of these methods are without risks and limitations. An alternative option to increasing biomechanical resistance to the pull-out effect is to tackle bone regenerative capacity and focus on screw osteointegration properties. Osteointegration was reportedly enhanced through various optimization strategies, including use of novel materials, surface modification techniques (e.g., application of coatings and topological optimization), and utilization of composites that allow synergistic effects between constituents. In this context, this paper takes a comprehensive path, starting with a brief presentation of spinal fixation devices, moving further to observations on how the pull-out strength can be enhanced with existing methods, and further focusing on techniques for implant osteointegration improvement.

## 1. Introduction

Spinal disorders are among the most common medical conditions, covering a wide range of pathologies [1]. The increase in life expectancy has left the population exposed to different degenerative changes and deformities in the aging spine [2,3]. Moreover, various daily habits, posture conditions, comorbidities, and traumas are reflected in the health of the spinal column [4]. Management of spinal disorders may comprise physical examinations, plain radiography, computed tomography scans, and magnetic resonance imaging to correctly and efficiently assess the diagnosis, establish the treatment plan, and determine the prognosis [5]. 

Despite most patients being treated conservatively, a significant and growing part of the population with spinal issues is subjected to surgical interventions [1]. Surgical spinal procedures have been practiced since Jules Gerin first attempted surgical scoliosis correction in 1839, while the employment of fixation devices was introduced by Dr. Berthold Earnest Hadra in the late 1800s as a mean of stabilizing C6–7 dislocations with a silver wire [6]. Since then, ensuring the mechanical stability of the spine has become a must for obtaining successful surgical outcomes, with scientists developing more and more varied and effective orthopedical implants [2,7,8]. Utilizing internal fixation systems, such as screws, rigid rods, plates, wires, and cages, is vital for stabilizing the spine’s structure, restoring its endurance, and protecting its function [2,5,6,9,10].

With spinal instrumentation at hand, spinal surgery represents a challenging treatment approach for spinal diseases, supposing the utilization of complex spinal fixation constructs and prolonged operative times. Moreover, performing spinal surgery on patients with osteoporosis is even more difficult, as these people are prone to perioperative complications which may also result in mechanical failures, such as screw pull-out or loosening [7,9,11,12]. A proper selection of fixation devices is mandatory to avoid procedure failure and eliminate the necessity of revision surgeries. Ideally, involved implants should possess biostability, biocompatibility, and appropriate biomechanical properties (e.g., Young’s modulus, stiffness, fatigue) [6]. 

In this context, this narrative review aims to briefly present the most used spinal fixation devices, further focusing on how the pull-out effect can be reduced through various techniques. Enhancement of the osteointegration capacity of implants is particularly discussed as a promising method for improving the pull-out strength of fixation devices, and several material optimization possibilities are analyzed. For the completion of this paper, we have analyzed recent English language publications from Science Direct, PubMed, MDPI, and Google Scholar databases, retrieved through various combinations of keywords, including “spinal fixation devices”, “spinal screws”, “pedicle screws”, “spine surgery”, “pull-out effect”, “osteointegration”, “reinforcement strategies”, “improved biomechanical properties”, and “material optimization”.

## 2. Spinal Fixation Devices

Spinal disorders necessitating internal fixation may be treated using several devices, among which plates, cages, rods, and screws are the most commonly employed [6,13,14]. Often used in various combinations, spinal fixation devices aid in holding the vertebrae together as one solid unit [13]. Consequently, such internal fixation systems are responsible for stabilizing spine structure, restoring its endurance, and ensuring its functionality [10]. To achieve these roles, they must be made of biocompatible or biologically inert materials with a Young’s modulus similar to that of natural bone, high tensile strength, stiffness, fatigue strength, and low artifacts on imaging. In this respect, the most frequently involved materials include titanium, stainless steel (SS), cobalt chrome (CoCr), nitinol (a nickel–titanium alloy), tantalum, and polyetheretherketone (PEEK) [6]. To shed some light on the most commonly used spinal fixation devices, the following subsections briefly describe the uses and main features of plates, cages, rods, and screws.

### 2.1. Plates

Metal plates represent essential implants for stabilizing the spine and restoring its normal alignment. Most of these devices are made of pure titanium (PTi) or titanium–aluminum–vanadium (Ti6Al4V), while other plates may also incorporate a polyethylene ring (used as a screw-locking mechanism within the plate). More recently, technological advances have led to the consideration of biodegradable materials for fabricating spinal plates [6]. In particular, in vitro comparison tests revealed that strong biodegradable plate and bone block (PLA-4G)-based plates offer similar motion, torque strength, and von Mises stress results as titanium, while possessing a much lower Young’s modulus. Moreover, lacking paramagnetic properties, the biodegradable plate produces fewer imaging artifacts [15]. 

Anterior plate fixation can be utilized for performing anterior cervical decompression and fusion (ACDF), ensuring immediate cervical spine stability with a reduced risk of pseudarthrosis. However, certain complications have been linked to plate fixation, including breakage, triangle fracture, utilization of screws that may loosen or penetrate the endplate, and trachea–esophageal or neurovascular structural injuries, especially when used for multilevel ACDF. To bypass these drawbacks, it has been proposed to use stand-alone cages that present self-locking anchoring clips to replace the roles of plates and screws [16].

### 2.2. Cages

Cages are spinal implants that aid in stabilizing force distribution between vertebral bodies, re-establishing the normal height of the intervertebral and foramina space, and furnishing support for vertebrae to re-fuse and heal when an intervertebral disc has failed. They originally started being used as a safer alternative to autologous bone grafts that exhibit issues correlated with donor site morbidity and high failure rates from collapse, subsidence, retropulsion, or resorption of the graft with prolonged healing time [6]. 

In the mid-1990s, cages assumed a cylindrical design, fabricated from threaded titanium alloy filled with autologous bone grafts. Despite their greater fusion rates than bone grafts and non-threaded cages, these devices were reportedly less stable in extension and flexion and more prone to cage subsidence. Since the early 2000s, non-threaded box-shaped titanium or PEEK interbody cages have been more prevalent due to their greater cage stability during flexion, axial rotation, and bending [17]. 

**Figure 1:**
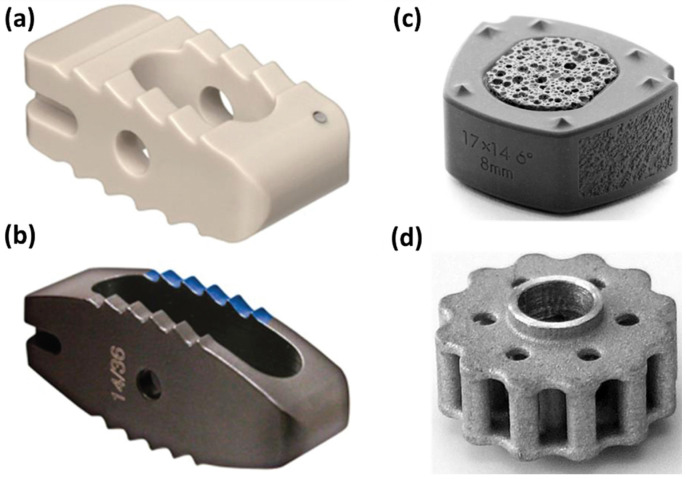
Visual representation of several interbody cages made of different materials: (**a**) PEEK cage; (**b**) titanium cage; (**c**) composite silicon nitride cervical implant; (**d**) skeleton of a magnesium-polymer cage. Adapted from an open-access source [17].

Ideally, an interbody cage should be rigid enough to ensure the necessary stability, but with a Young’s modulus similar to that of bone to avoid subsidence and stress-shielding [18]. Typical cage materials currently used suppose PTi, titanium-based composites and alloys, ceramics, or plastics [6] (Figure 1). Among these, titanium and its alloys remain the pillar for manufacturing interbody spinal cages due to their advantageous properties (e.g., ability to enhance cell adhesion and osteointegration, superior structural properties, high corrosion resistance, and low density). Nonetheless, one major problem with titanium is its unmatched elasticity modulus with cortical bone that might cause cage subsidence, further resulting in screw loosening, cage migration, non-union, rod breakage, and necessitating revision surgery [18]. Another highly employed material for fabricating vertebral interbody cages is PEEK. PEEK benefits from low manufacturing costs, bioinertness, radiolucency, and an elasticity modulus comparable to that of cortical bone. In contrast to titanium, PEEK cages have a lower subsidence rate and are MRI-compatible. However, PEEK surfaces provoke the development of fibrous tissue instead of stimulating osteogenesis. To solve this issue, PEEK cages are generally packed with bone grafts to accomplish spinal fusion [17,19,20]. Differently, porous materials have been reported to support bony healing into the cage itself, and the use of metal alloys and/or ceramic materials represents a valuable alternative [19]. In recent years, the development of bioabsorbable cages has also been tackled, with the use of magnesium-polymer fixation devices being explored as implant options. Nevertheless, such cages do not promote osteointegration. Therefore, this area should be extensively researched before achieving constructs that degrade with time, encourage osteointegration, and also possess a bone-like elastic modulus [17]. 

### 2.3. Rods

Rods are spinal fixation devices used in all spinal segments, ranging from cervical to sacral regions and including both the anterior and posterior spine [21]. Rods are frequently employed in treating spinal deformities as an efficient posterior fixation method for restoring the physiological spine curvature and maintaining a well-balanced condition [22]. In this respect, rods are bent when they are implanted within the tiny space inside a patient’s body to attain the in situ spine profile [23]. Even though preformed spinal rods are available on the market, intraoperative contouring is preferred as it allows surgeons to ensure a satisfactory matching with a patient’s spinal curvature [22]. 

Regarding biomechanical properties, rods must be made from materials with a sufficiently high Young’s modulus to suppress spring-back (i.e., reverse bending) and sufficiently low to prevent stress shielding [24,25]. Therefore, it should be possible to increase the rigidity of the bent parts of the spinal fixation device by room temperature deformation, while the remainder of the rod would remain unchanged at a low rigidity value. Titanium alloys were often employed to satisfy these requirements, given their excellent biocompatibility and adaptable rigidity [23]. Cobalt–chromium rods also present satisfactory properties. Compared to titanium, CoCr devices are better for the effective correction of spinal deformity and postoperative stability of the spine but are linked to higher risks of proximal junctional kyphosis [5]. Magnetically controlled growing rods can be utilized for the early onset of scoliosis. However, the complexity of these implants renders them more prone to succumb to multifarious failure modes than conventionally growing rods [26].

### 2.4. Screws

**Figure 2:**
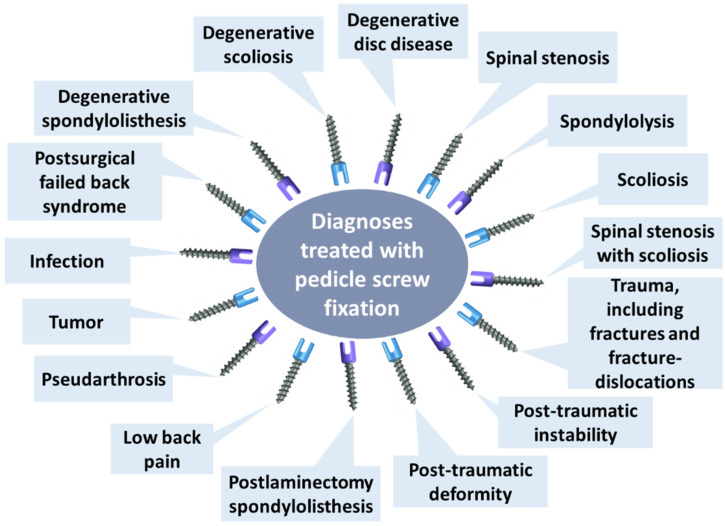
Diagnoses that can be treated with pedicle screws. Created based on information from [28].

Pedicle screws represent common implantable devices for fixing spinal vertebrae, given their insertion through all three columns of the vertebrae that ensures a rigid attachment of the spine [14,27]. Since their introduction in clinical practice in 1969 and further development in the late 1980s, pedicle screws have been proven valuable in treating numerous spinal diagnoses (Figure 2), becoming standard instrumentation for spinal surgery [27,28]. Spinal fixation with pedicle screws is frequently employed in stabilizing fractures and dislocations in the thoracolumbar region, correcting deformity or instability, and fixing oncological and fusion procedures [29,30]. Moreover, because pedicle screw systems do not necessitate intact dorsal elements, they can be utilized after a laminectomy or traumatic disruption of laminae, spinous processes and/or facets [14]. 

The inclusion of pedicle screws in the instrumentation of the fusion procedure was noted to increase the initial stability and the probability of achieving a successful spinal fusion in the fusion segment [31]. Other advantageous features of pedicle screw systems include their firm fixation, ability to restore the normal physiological radian of the spine and the volume of the spinal canal, effectiveness in achieving complete decompression and relieving the strain on the spinal nerves, provision of a favorable environment for the recovery of nerve function, and early patient discharge [30]. 

To successfully accomplish its role, a pedicle subassembly must have a high strength under all possible conditions [14]. Specifically, pedicle screw systems must endure intraoperative loading and physiological forces caused by daily postoperative activities [32]. Hence, a strong bioinert material, such as Ti6Al4V, is required for their manufacture [6]. 

### 2.5. Overview

To offer a brief perspective of the above-discussed spinal fixation devices, Table 1 summarizes the applications, standard fabrication materials, advantages and disadvantages of each category. 

Several spinal implants are used in clinical practice for restoring spine stability, correcting deformities, and ensuring proper fixation in various health conditions. Depending on their role, placement, and treated disorder, spinal fixation devices must be made from specific materials endowed with suitable biomechanical properties. However, as a general rule, optimal outcomes in the fused segment and fewer side effects in the adjacent levels are achieved when using biofunctional and biologically inert/biocompatible, less rigid systems of fixation. Involved Biometals should consist of nontoxic and nonallergic elements, should be corrosion-resistant to avoid the dissolution of metallic elements, and possess appropriate mechanical properties. An ideal biomaterial for spinal fixation devices must exhibit high fatigue strength, stiffness, low artifacts on imaging, and similar-to-bone rigidity while maintaining a strength above that of bone until complete fusion is achieved [6,21,23,31]. These characteristics are challenging to accomplish with a single material, and increasing interest has shifted to developing plates, cages, rods, and screws made of various alloys and composites that can synergically combine the advantages of several components. 

## 3. Pull-Out Effect

Spine stabilization devices are essential instruments in managing spine deformities, degenerative diseases, and trauma. The design, development, and testing procedures have been documented in the literature, establishing the influence of thread depth, outer diameter, bone quality, insertion technique, and augmentation technique on the strength of the bone–implant interface based on the pull-out tests [37]. 

**Figure 3:**
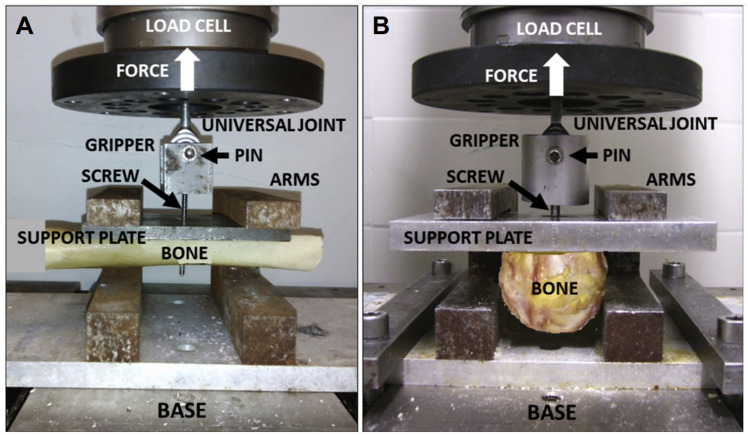
Bone screw pull-out tests. (**A**) Cortical screw pull-out test using the cortical midshaft segment of a human long bone, (**B**) cancellous screw pull-out test from the cancellous bone of the proximal head or distal condyle removed from a human long bone. Reprinted with permission from [38], Copyright Elsevier, 2017.

Pull-out tests are important in vitro experiments for determining the biomechanics of screw–bone interactions and failure forces [32]. They are performed by applying an axial force at a constant displacement rate to a pedicle screw previously introduced into a cadaver or synthetic bone sample, measuring the maximum force required before the screw loses its fixation [37] (Figure 3). 

Screw pull-out is one of the main device failures, especially if such implants are placed in low-density osteoporotic bones or are subjected to high loads (as is the case of fixation of fractures of the femoral neck or the lumbar spine) [39,40]. Additionally, hardware loosening or pull-out can be provoked by micro-motion, injuries, or excessive stress concentrations at the bone–implant boundaries, leading to structural failure of fixation systems [41,42]. Correction of misaligned posterior instrumentation may also cause screw pull-out and high-tissue strains in adjacent and downstream spinal segments [43]. Moreover, when a pedicle screw is pulled out, it leads to fractures of the bone between the crests of the thread [44]. 

In this context, various improved designs and augmentation techniques had to be developed to increase screw pull-out strength and avoid consequential device failure. Specifically, the biomechanical characteristics of the bone–screw interface can be enhanced by several methods, such as increasing outer screw diameter, employing a cortical bone trajectory, using bone cement, undertapping, and utilizing expandable pedicle screws [29,40,44,45,46]. 

Patel et al. [47] have investigated how pull-out strength is affected by the insertion angle in normal and osteoporotic bone models of screws made of titanium alloys. The researchers reported that screws inserted at 0° and 10° in a normal cancellous bone model exhibited larger pull-out forces compared to screws inserted at 20°, 30°, and 40° angles, while for the osteoporotic bone model, no trend was identified. 

Alternatively, several studies have been centered around various size parameters, investigating their influence on the pull-out strength of screws. According to Bianco et al. [32], screw diameter has an important influence on anchorage performances as this feature is directly related to crest height and, subsequently, to the contact surface with the bone. Karakaşlı et al. [48] observed that non-locking screws with larger diameters and pitch depth demonstrated superior performance than locking screws with smaller dimensions, requiring larger pull-out forces for their extraction. It was also reported that using screws with increased diameter and length inserted through an undertapped pilot hole smaller than the core diameter of the screw may aid in increasing pull-out strength [41]. However, the authors concluded that larger screws do not affect the fixation strength in osteoporotic bone, given the thin cortex of the pedicle. On the other hand, a study performed by Varghese et al. [49] revealed that, in revision surgeries, using screws 2 mm in diameter greater than the original screws increases pull-out strength by 125%. 

Even though larger diameter screws improve pull-out strength, oversizing is not indicated, as it may result in pedicle fracture of the osteoporotic vertebrae [50]. Alternatively, the length of the screw can be expanded to improve resistance to pull-out forces [27]. For instance, tests performed by Conrad and colleagues [51] demonstrated that each 1 mm increase in screw length leads to a 16 N increase in the pull-out force. Scientists have also indicated that screws with higher depths increase the contact area of cortical and cancellous bone, reflected in an improved pull-out strength [52]. 

Furthermore, hybrid posterior constructs combining pedicle screws, sublaminar wires, and laminar hooks can raise pull-out strength in osteoporotic bone, boosting overall construct stability [12]. This is because mixing several fixation techniques helps distribute stresses on the osteoporotic spine. For example, combining hooks and pedicle screws (i.e., pediculolaminar fixation) was noted to increase the pull-out strength up to 100%, being also able to heighten stiffness of the system and increase torsional stability in osteoporotic bone. Nonetheless, clinical evidence to support these augmentation techniques is limited by the technical challenges in connecting these supplemental fixation points to the rods between the screws [41]. 

Important observations have also been made concerning the relation between screw profile (conical vs. cylindrical) and screw performance [32]. Studies performed by Chao et al. [53] revealed a higher pull-out strength in screws with a conical core and smaller core diameter. Amaritsakul and colleagues [54] attributed the pull-out strength of the conical design to the bone compaction effect. Moreover, the authors reported that even greater stability could be achieved by dual inner core screws and double dual-core screws, while extra care should be taken when using a cylindrical screw with a small thread depth as it may easily break around the thread shank region. In contrast, Kim et al. [55] registered the highest pull-out strength for V-shaped thread screws with an outer cylindrical and inner conical shape, regardless of bone density. Similarly, Martin-Fernández et al. [3] recorded increased pull-out strength when using screws with standard or V-shaped threads with a larger diameter and better fixation at the pedicle.

**Figure 4:**
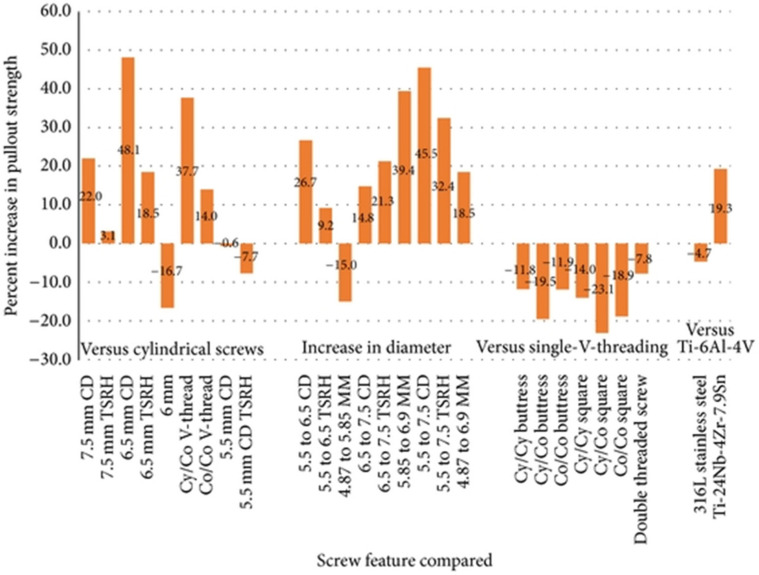
Percentage difference in pull-out strength produced by screw design variation compared to a classic pedicle screw of otherwise similar dimensions. Abbreviations: TSRH—Texas Scottish Rite Hospital (conical screw), CD—Cotrel–Dubousset (conical screw), MM—Moss Miami (cylindrical screw), Cy/Cy—cylindrical thread with cylindrical core, Cy/Co—cylindrical thread with conical core, Co/Co—conical thread with conical core, V—standard thread, and Ti—titanium. Adapted from an open-access source [56].

Offering a quantitative perspective to pull-out strength increase by varying screw design, Shea et al. [56] have compared literature data on several features, as depicted in Figure 4. 

Modifications to standard pedicle screw design can also achieve improvement of screw stabilization in a way that compensates for the detrimental effects of decreased vertebral bone density. Specifically, expandable titanium pedicle screws can potentially improve pull-out strength, especially for managing traumatic and degenerative spinal diseases in osteoporotic patients [12,40,57,58].

Several studies also focused on the influence of tapping insertional torque as it may allow tactile feedback for optimal screw size selection and improved pull-out strength. Explicitly, undertapping the pedicle screw tract was noted to increase pull-out strength [12,50].

Another promising strategy to diminish the pull-out effect is bone cement infusion, which has two-fold applicability: analgesia and internal fixation [59]. One of the most commonly employed cement augmentation methods is the polymethylmethacrylate (PMMA) injection. PMMA significantly increases the axial pull-out strength of augmented pedicle screws in both primary and revision procedures, being a valuable technique for reinforcing the bone–screw interface [9,40,60]. Despite being widely used in orthopedic surgery, PMMA cement augmentation had a delayed adoption in spinal surgery due to a series of risks. The main problems with using this type of cement are the possibility of thermal necrosis caused by the exothermic polymerization reaction of PMMA, the risk of neural injury in case of polymer extravasation, and challenges in performing revision surgery [9,40,41,61]. In more detail, the leakage of PMMA into the spinal canal may produce permanent thermal damage to the spinal cord, while the non-biodegradable nature of this polymer translates into a lasting stenosing mass left in the spinal canal in the event of extravasation [40]. It has also been hypothesized that cement can embolize into the venous system through the epidural veins and reach the right atrium and pulmonary arteries, eventually leading to right heart strain or respiratory failure [61]. Thus, increasing interest is expected in developing safer biodegradable cements with similar reinforcing properties. 

**Figure 5:**
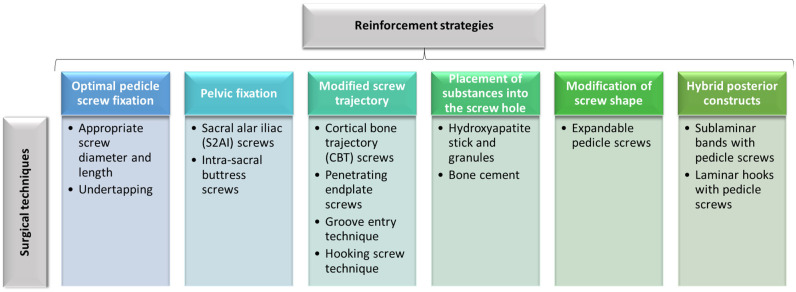
Surgical techniques to reinforce screw fixation in patients with osteoporotic spine. Created based on information from [50].

Thus, surgeons should choose screws of appropriate size and shape and thoroughly plan the surgical strategy for optimal outcomes. Moreover, to ensure better spinal stability and rigid screw fixation, clinicians can address the use of various reinforcing techniques that are summarized in Figure 5 [50]. 

## 4. Osteointegration

Osteointegration (also found in the literature as “osseointegration”) represents a dynamic and complex biological process ruled by protein absorption on the implant surface immediately after its insertion [62]. Assuming the ingrowth of the implant into the bone, osteointegration creates a direct bond to the bone resulting in a firm anchorage [63]. Hence, improving the osseointegration properties of fixation devices holds promise for enhancing their biomechanical behavior toward avoiding the pull-out effect.

**Figure 6:**
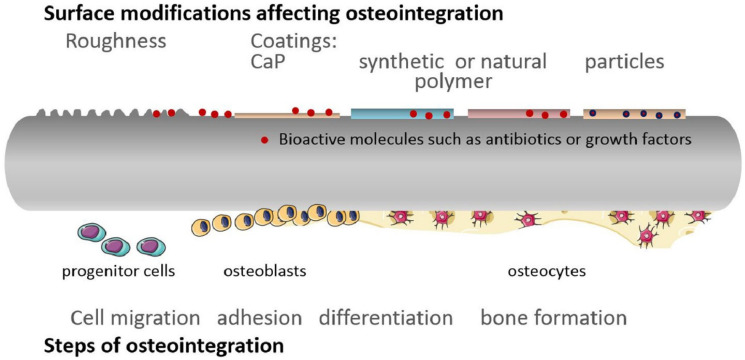
Schematic representation of the osteointegration steps and the main implant surface modification strategies for promoting this process. Reprinted from an open-access source [63].

Osteointegration was noted to depend on surface topography, chemical composition, hydrophilicity, and surface roughness, being also in close connection with osteoconduction, osteoinduction, and osteogenesis processes [62,63,64] (Figure 6). Osteoconduction concerns the process of directing the bone formation on a particular site or surface (e.g., hydroxyapatite-coated surface, bioglass, glass–ceramic) by directly bonding to the living bone by apatite. On the other hand, osteoinduction supposes the recruitment of mesenchymal stem cells and growth factors to induce the osteogenesis microenvironment even if the implant is placed into an ectopic site [62].

In the first stages of osteointegration, proteins from the blood and serum cover the implant surface according to the chemical and topographical nature of the device. Further, this protein coating promotes the migration of mesenchymal progenitor cells into the implant surface via the α2β1 integrin receptor, creating a proper environment for differentiation into osteoblasts that are needed for new bone formation. This process is also influenced by the surface properties of the implant, with osteoblastic differentiation being supported by rough facets [64]. Moreover, implant surface topography affects the cytoskeletal arrangement of primary osteoblast cells, influencing their functions both in vitro and in vivo [65]. 

With these aspects in mind, researchers investigated various surface-modifying strategies for fixation devices, including implant roughening and coatings application. Coatings can be embedded with different active factors (e.g., antibiotics, growth factors, nanoparticles) to ensure better osteointegration of the fixation system [63]. For instance, even if titanium is known to be inert, through specific chemical and thermal surface treatments, its osteoconductivity can be improved to enhance bonding to living bone [62]. Moreover, it has been reported that roughened titanium can generate an osteoblastic environment, whereas PEEK inhibits it, leading to the development of an inflammatory fibrous rind. In addition, the utilization of nanoscale surface technology allows host cells to interact with the implant at a molecular level through cellular membrane receptors, triggering osteoblastic-lineage differentiation and boosting fusion outcomes [64]. 

Novel evidence in the field also suggests that surface topographical cues are vital for speeding osteointegration when the osteoimmune tissue microenvironment is properly modulated. In more detail, osteoimmunomodulation governs osteointegration through the mediation of immune cell response and subsequent bone cell behaviors. Specifically, after insertion, the innate immune system becomes active at the interface between the implanted biomaterial and tissue within hours, creating an immune environment adjacent to the fixation device. Therefore, biomaterials that exhibit a favorable immune response can also result in a more successful osteointegration and improved fixation [66].

## 5. Material Optimization

Most orthopedic screws are made of biocompatible metallic alloys that are stiffer than natural bone and produce lacerations to the tissues, producing strong inflammation [67]. Titanium alloy-based implants are also disadvantageous because of the association with artifacts on postoperative imaging, impeding radiation treatment planning, execution, and follow-up imaging [68]. 

In this context, research efforts have been directed toward optimizing the materials used for fabricating spinal fixation devices, especially to improve their osteointegration ability in order to increase screw stability and pull-out strength [69]. Noteworthy reports evidenced the potential of coating metallic surfaces with bioactive layers to promote osteointegration, stimulate bone growth, and enhance bonding strength at the implant interface. For instance, carbon-based materials represent suitable options for coating titanium implants, promising to improve bioactivity and osteointegration [62]. Surface modification with hydroxyapatite, metallic oxides, or polymer was also investigated to improve spinal fixation devices’ performance [63]. In particular, Yadav et al. [67] have suggested the use of a metallic core (i.e., Ti6Al4V) and polymeric shell (i.e., poly L-lactic acid) orthopedic screws, reporting an improved structural rigidity for the composite device.

Bioceramics, such as calcium sulfate, hydroxyapatite, and calcium silicate, have been the object of research in many bone regeneration studies as they are known to accelerate mineralization while also being endowed with low cytotoxicity, high bioactivity, and excellent biocompatibility. Moreover, their microstructure promotes ossification and vascularization, which are essential steps in the broader processes of osteointegration and osteoinduction [70,71]. For example, calcium phosphate cements are an attractive alternative to PMMA. These bioceramic materials possess a compressive strength between that of cancellous and cortical bone but exhibit shear and tensile strengths below those of cancellous bone. Despite producing a lower increase in pull-out strength than PMMA, calcium phosphate cements still benefit from a greater bending rigidity and stiffness than non-augmented screws [72]. 

Another highly valuable material in bone regeneration applications is hydroxyapatite. Its appealing properties include biocompatibility, porous structure, high chemical and crystallographic similarity to the inorganic phase of native bone, capacity to bind to natural bone, and ability to enable the growth of surrounding tissues [73]. With these advantages in mind, Jang et al. [74] tested hydroxyapatite cement for screw augmentation in spinal osteoporosis, obtaining encouraging results. More recently, Ślósarczyk and colleagues [75] have created biomicroconcretes containing porous hydroxyapatite–chitosan granules and α-tricalcium phosphate-based bone cement. The authors reported similar compressive strength to spongy bone and promising physicochemical properties for their composite materials that should, nonetheless, be further confirmed through biological tests. 

On a different note, Müller and Djurado [76] have newly developed a series of composite materials consisting of bioactive glass and hydroxyapatite as coatings for improving the overall osteointegration of Ti6Al4V-based implants. The thinnest composite coating gave the best result, demonstrating better mineralization performance than its thicker counterparts and single-material coatings. An interesting approach is also proposed by Honda et al. [77], who have fabricated a protamine-loaded hydroxyapatite coating. Researchers reported that the developed materials were biocompatible, osteoconductive, and bactericidal, inhibiting adhesion and early growth of bacteria at the material’s surface while promoting osteointegration. 

Advances in material optimization have also tackled the creation of different metallic alloys with improved properties. For instance, aiming to overcome poor osteointegration and loosening risk of standard titanium-based implants, Zhang et al. [78] have fabricated screws from copper-alloyed titanium. The authors tested the new class of implants on osteoporosis-modeled rats, obtaining encouraging results. Specifically, the novel alloy could stimulate vascular network reconstruction around the implant by upregulating vascular endothelial growth factor expression and further promoting the proliferation and differentiation of osteoblasts, mineralization, and deposition of collagens, considerably enhancing bone mineral density within the peri-implant area. As it improved fixation stability, accelerated osteointegration, and reduced aseptic loosening risk, the alloy was concluded to be a potential candidate for future fixation devices in osteoporotic patients. 

Given the importance of PEEK material in spinal fixation applications and its weak osteointegration properties, several studies have also investigated methods to improve the performance of PEEK-based devices [79]. One appealing strategy is the use of carbon-fiber-reinforced PEEK pedicle screws and rod fixation systems for spinal tumor surgery [80]. Ringel et al. [68] have reported their experience in using a carbon fiber-reinforced PEEK pedicle screw-based internal fixator in managing spinal metastases and primary spinal tumors. The authors used screws containing endless carbon fibers in 55% volume embedded in a PEEK matrix, and for enhanced bone integration they coated the area of the pedicle with titanium. In vivo studies confirmed an increased turnout resistance of titanium-coated screws, leading to the hopeful conclusion that these new internal fixators might be superior in long-term stability due to less material fatigue, as compared with standard titanium alloy devices. Another possibility is the use of polyetherketoneketone (PEKK) instead of PEEK, as this material possesses more ketone groups and better potential for surface chemical modification. This method was suggested by Yuan et al. [79], who compared various PEEK and PEKK-based materials, showing easier sulfonation, better bone-like apatite deposition, enhanced osteointegration, and improved mechanical stability for PEKK. 

On another note, Raj et al. [8] have recently proposed using biodegradable orthopedic implants. In this respect, the authors produced 3D-printed poly(lactic acid) models and further functionalized their surfaces with TiO_2_ + ZrO_2_ nanocomposites via the dip-coating method. In vitro tests revealed promising outcomes, as osteointegration was observed to improve as the number of days of treatment was prolonged, indicating the improved mechanical properties of the developed constructs and their potential for orthopedic applications. 

Topological material optimization methods have also been noted to generate highly efficient fixation devices. Through metal additive manufacturing techniques, Cucinotta and colleagues [81] have optimized a femoral nail to allow a less invasive intervention and improve patient comfort. However, further studies are needed to establish osteointegration outcomes and translate this approach to spinal fixation devices.

Interesting possibilities can also be envisaged by employing nanotechnology surface features for increasing osteointegration and diminishing screw pull-out [64]. For instance, Divya Rani et al. [65] have fabricated metallic titanium implants with various tunable non-periodic nanostructured surfaces. Among the tested nanomorphologies, the nanoleafy pattern demonstrated the highest impact on protein adsorption, in vitro osteoblast cell proliferation, and differentiation. In vivo tests also revealed that these nanostructured implants exhibited a higher percentage of bone contact without any inflammatory response, confirming the importance of specific nanomorphologies in controlling tissue integration.

Table 2 was created to enhance the clarity of the discussion and provide more insight into the described material optimization studies. 

In terms of fabrication methods, novelty arises from additive manufacturing. Such technologies allow the development of implants either as solid structures or as constructs composed of numerous interconnected open-pore architectures. Compared to conventionally machined screws, fixation devices obtained through these methods benefit from a micro-rough surface that improves bone growth [62]. Additive manufacturing techniques are also effective for producing porous cages of well-controlled morphology [23].

## 6. Conclusions

Spinal fixation devices are essential to ensuring spine stability and aiding in the healing process of various spinal disorders. However, most orthopedic implants are made of metallic materials stiffer than natural bone, are prone to the pull-out effect, and may fail over time, requiring revision procedures. In this respect, reinforcement strategies, such as modification of screw trajectory, adapting screw shape, injecting bone cement, and utilizing hybrid constructs, are employed in clinical practice, each with its risks and limitations.

To further improve the biomechanical resistance of spinal screws, osteointegration of the implant has been proposed as an appealing strategy. Since bone integration properties have reportedly been associated with surface implant features, several surface modification strategies have been proposed to enhance the fixation systems’ pull-out strength. The main osteointegration improvement approaches include roughening implant surfaces, creating nanostructured surfaces, and applying bioactive coatings. Yet, most of these possibilities have only been tested through in vitro and in vivo studies and require more in-depth evidence before being translated to the clinical setting. In conclusion, despite the emergence of several directions for improving the biomechanical properties of spinal fixation devices, more research is needed to confirm the efficacy of these possibilities under human clinical conditions.

## Figures and Tables

**Table 1:** Overview of applications and materials of spinal fixation devices.

Devices	Applications	Standard Materials	Advantages/Benefits	Disadvantages/Problems	Refs.
Plates	Spinal stabilization	Titanium	Increased spinal stability Direct decompression of the spinal cord High rate of neurologic improvement Low complication rate	Not preferred for multilevel fusion Not a stand-alone fixation method	[6,33,34]
Cages	Anterior/posterior interbody fusion (restore the height of collapsed disc from injury, degenerative disc disease, or scoliosis)	Titanium PEEK Ceramic Acrylic	Increased spinal stability Preserved facet joints Minimal destruction of the posterior and facet joint ligaments and of the endplates	Fibrosis occurrence Increased minimal principal stresses Maintenance of disc heights and lordotic alignment not achieved in the long term	[6,35]
Rods	Spinal fusion (add stability to a spinal implant; used for scoliosis correctional surgery)	Titanium CoCr PEEK Stainless steel Nitinol	Increased spinal stability High fusion rates Increased load sharing Preferred over plates for multilevel fusion	Not a stand-alone fixation method	[6,34,36]
Screws	Pedicle screw fixation (holds vertebrae together to attach plates and rods)	Titanium (Ti6Al4V) doped with:	Increased spinal stability Firm fixation Complete decompression and strain relief on the spinal nerves Early patient discharge	Loss of fixation Improper placement Fatigue and bending failure Dural tears Cerebral spinal fluid leaks Nerve root injury Infection	[6,30,31]
		HA CaP ECM Tantalum			

Abbreviations: PEEK—polyetheretherketone; Ti6Al4V—titanium–aluminum–vanadium; HA—hydroxyapatite; CaP—calcium phosphate; ECM—extracellular matrix; CoCr—cobalt–chromium alloys.

**Table 2:** Summary of discussed material optimization strategies for osteointegration enhancement.

Proposed Strategy	Device Specifications	Observations	Ref.
Screws with metallic core (i.e., Ti6Al4V) and polymeric shell (i.e., poly L-lactic acid)	Pull-out force: 150–182 N Bending force: 574–614 N	Improved structural rigidity Quick healing property for the bones attributed to the outer bioabsorbable material Minimization of the loading on the bone during dynamic activities	[67]
Biomicroconcretes containing porous hydroxyapatite–chitosan granules and α-tricalcium phosphate-based bone cement	Total open porosity: 45 ± 5 vol.% (Series A) and 50 ± 5 vol.% (Series B) Pore size distribution: ranging from 0.005 µm to 40 µm Mechanical strength of Series A composites: ranging from 5.4 ± 0.8 MPa to 6.2 ± 1.0 MPa [similar to compressive strength of trabecular bone (4–12 MPa)]	Mechanical strength is influenced by the amount of chitosan in hybrid HAp–CTS granules Higher compressive strength was noticed for biomicroconcretes containing granules with a lower amount of chitosan (17 wt.%)	[75]
Bioactive glass/hydroxyapatite composites for Ti6Al4V implant improvement	Morphology: highly porous coral-like coating Bioactive glass thickness: ranging from 6 μm to 30 μm Nanocrystalline hydroxyapatite layer thickness: 150 nm	The presence of the bioactive glass-topcoat layer significantly improves the reactivity in terms of mineralization response compared to single-layered hydroxyapatite coating	[76]
Antimicrobial protamine-loaded hydroxyapatite coating	Disc diameter: 15 mm Disc thickness: 1–2 mm Antimicrobial powder amount: 0.30 g	Bactericidal properties against *Escherichia coli* and *Staphylococcus aureus* Osteoconductivity and biocompatibility proven through in vitro and in vivo tests	[77]
Copper–titanium alloy screws	Tensile strength: 597 ± 3.1 MPa Elongation: 26% ± 3.5% Yield strength: 457 ± 7.0 MPa Vickers hardness: 215 ± 8.5 HV Total length: 7 mm Upper width: 4 mm Thread width: 2 mm Thread length: 3 mm	Excellent mechanical properties and bio-functionalization Corrosion resistance and antibacterial performance Improved fixation stability stimulates the vascular network reconstruction around the implant Promotes the proliferation and differentiation of osteoblasts, mineralization, and deposition of collagens	[78]
Radiolucent carbon fiber-reinforced PEEK pedicle screw coated with titanium	Carbon-fiber amount: 55% volume Length: 100 mm Diameter: 5.5 mm	Increased turnout resistance Less material fatigue than standard titanium alloy devices Postoperative artifact–reduced imaging	[68]
Porous PEKK implants	Morphology: interconnected macropores and micro/nano topography Pore size: >200 μm Maximum push-out force: 97.6 ± 9.4 N	More than double the amount of newly formed bone than in PEEK implants Better osteointegration and mechanical stability than PEEK devices	[79]
Biodegradable poly(lactic acid) implants surface-functionalized with TiO_2_ + ZrO_2_ nanocomposites	Crystal size of Ti–Zr nanocomposites: 19.2 nm Nanocomposite layer weight: ~62 mg Weight of apatite layer deposited on the 18th day: 65 mg	Improved osteointegration Enhanced mechanical properties	[8]
Metallic titanium implants with nanoleafy surface pattern	Surface morphology: a network of vertically aligned, non-periodic, leaf-like structures with thickness in the nanoscale Critical load to adhesive failure: 0.44 ± 0.023 N	Good biocompatibility A higher increase in osteoblast cell proliferation, alkaline phosphatase activity, and collagen synthesis than other nanomorphologies A higher percentage of bone contact with no inflammatory cytokine production	[65]

## Data Availability

Not applicable.
